# Aphonia and epiglottitis in neonate with concomitant MRSA skin infection

**DOI:** 10.1002/rcr2.66

**Published:** 2014-08-25

**Authors:** Jennifer Noble, Renee Devor, Francis J Rogalski, Wilfredo Vergara, Ramalinga P Reddy, Nasreen Bhumbra

**Affiliations:** Department of Pediatrics, University of ToledoToledo, Ohio, United States of America

**Keywords:** Aphonia, bronchoscopy, deep venous thrombosis, epiglottitis, methicillin-resistant *Staphylococcus aureus*, neonate, Panton–Valentine leukocidin, supraglottitis

## Abstract

We report an unusual case of a neonate with aphonia due to epiglottitis with a concomitant methicillin-resistant *Staphylococcus aureus* (MRSA) infection of the genitalia and associated septic emboli to the groin area and mouth. We postulate that the MRSA infection caused a transient bacteremia that seeded the epiglottis, likely causing the epiglottitis. In the evaluation of a neonate with aphonia, while the two primary differentials to consider are vocal cord paralysis and laryngeal web, among other considerations is epiglottitis (supraglottitis).

## Introduction

Epiglottitis usually occurs in children aged 2–6 years with a peak incidence at age 3 years. Historically, epiglottitis has been caused by *Haemophilus influenzae* type b (Hib); however, in the post-Hib vaccination era, the incidence has dropped, allowing for a broader range of infectious agents including nontypeable *Haemophilus influenzae*, beta-hemolytic streptococci, staphylococci, and pneumococci [1], [2]. Unlike older children who present with stridor and respiratory distress with epiglottitis, the presentation of this disease in neonates may be different. There are some cases of acute epiglottitis caused by methicillin-resistant *Staphylococcus aureus* (MRSA) documented in adults confirmed by cultures of blood and tracheal secretions [3–5]. We describe a rare case report of epiglottitis likely caused by MRSA in a neonate.

## Case Report

A full-term Caucasian male was born by normal spontaneous vaginal delivery with birth weight of 3.4 kg. The mother was Group B streptococcus negative, with no additional reported infections or complications during pregnancy. The patient passed meconium within 24 h of life and was circumcised on day two of life. The patient was found later that day to have developed hyperbilirubinemia and was treated with triple phototherapy for 18 h. He was discharged home on day three of life and the mother noted that the patient had a hoarse cry at the time of discharge. He was fed breast milk supplemented with Similac Advance 2–3 oz every 3–4 h and was voiding and stooling well.

On day four of life, the patient still with a hoarse cry was taken to the primary care physician’s office and noted to have swelling and discoloration of the glans penis. He was referred the same day to the pediatric urology clinic. The urologist noted bladder distension, swelling on the glans with an eschar and moderate erythema of the scrotum. The meatus was black in color and stenotic with minimal urine expressed from the meatus. A 6 French Foley catheter was passed through the meatus into the bladder. The specimen sent for culture was found to be negative. The patient was sent home with the Foley catheter still in place. The patient returned to the urology clinic the next day with fever 38.5°C, increased fussiness, and decreased oral intake. The swelling on the glans now revealed a gun-metal gray eschar with moderate erythema of the scrotum with hematuria present. There were two vesiculo-pustular lesions 2–3 mm in diameter each with an erythematous base in the groin. The patient was admitted to the pediatric floor for evaluation for possible sepsis. Once cultures were sent for blood, urine, and cerebral spinal fluid, the patient was started on ampicillin 200 mg/kg/day and cefotaxime 200 mg/kg/day, both divided into 6 hourly doses.

**Figure 1:**
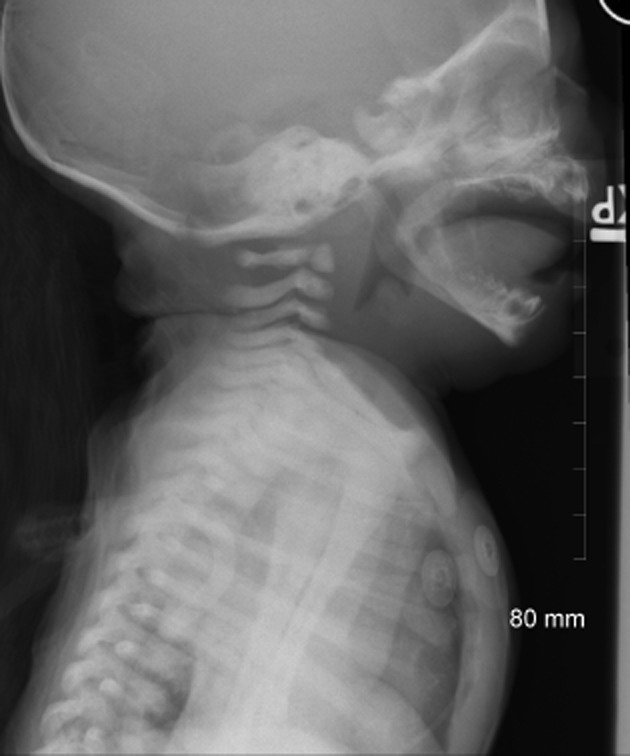
Soft tissue X-ray neck shows enlarged epiglottis and arytenoids, thick and convex aryepiglottic folds, and a distended hypopharynx consistent with epiglottitis (supraglottitis).

Shortly after admission to the pediatric inpatient floor, the patient became tachypneic, tachycardic, hypotensive, aphonic, and had decreased activity. He was transferred to the pediatric intensive care unit for further management. A lateral neck X-ray was obtained, which showed enlarged epiglottis and arytenoids, thick and convex aryepiglottic folds, and a distended hypopharynx consistent with epiglottitis (Fig. 1). Viral cultures of the rectum, nose, and mouth were obtained along with a Bordetella pertussis DNA by polymerase chain reaction (PCR) and the patient was started on azithromycin for empiric treatment of pertussis.

**Figure 2:**
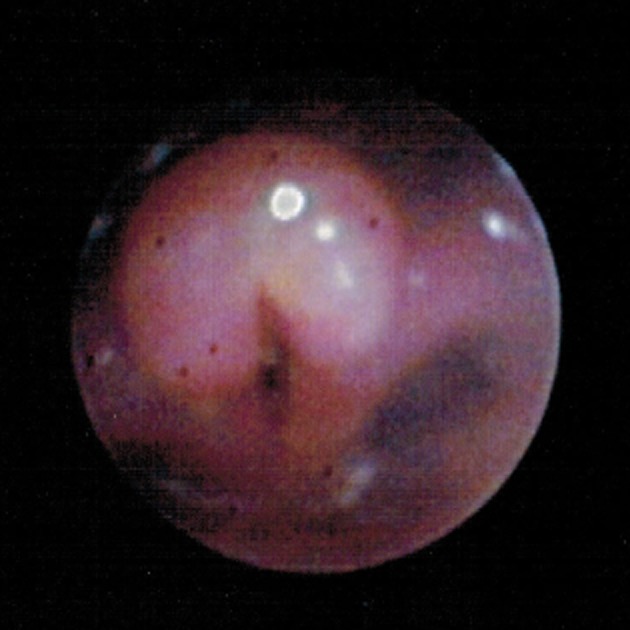
Bronchoscopy image shows a swollen and inflated epiglottis and arytenoids along with aryepiglottic folds consistent with acute epiglottitis (supraglottitis).

On day two of admission, a bronchoscopy was carried out using a flexible fiberoptic bronchoscope 2.8-mm Olympus XP160 (Olympus Medical Systems Corp., Tokyo, Japan) by a pediatric pulmonologist. The procedure showed right choanal stenosis, a swollen and inflamed epiglottis and arytenoids along with aryepiglottic folds consistent with acute epiglottitis (supraglottitis) (Fig. 2). Interestingly, there was no exudate present; therefore, cultures were not obtained directly from the epiglottis during this procedure. On day three, the patient developed several punctate ulcerative lesions on the tongue.

Due to the presence of septic emboli, a lower extremity venous ultrasound scan was obtained, which showed superficial phlebitis of greater saphenous veins and lesser saphenous veins but no thrombus. Immunodeficiency was also entertained with the multisystem presentation and additional studies were obtained. Immunoglobulin panel results included IgG 687 milligrams per deciliter (mg/dL) (241–870 mg/dL), IgA 7 mg/dL (1–52 mg/dL), and IgM 27 mg/dL (16–95 mg/dL) and complement levels C3 95 mg/dL (79–180 mg/dL) and C4 19 mg/dL (13–59 mg/dL). All results were within normal limits. The neutrophil oxidative burst test was negative. Immunologic studies in neonates are not as functional as in older children and adults, so these test results were marginally useful.

The straight catheter urine culture obtained on admission grew MRSA with > 100,000 colony forming units (cfu) per milliliter of urine. All cerebral spinal studies were negative. Two sets of blood cultures were obtained during the admission and both were negative. Skin culture of the eschar site on the groin grew MRSA moderate growth and a swab of the oral lesions grew MRSA light growth. MRSA sensitivities were as follows: clindamycin (S)/D-test negative, gentamicin (R), levofloxacin (intermediate), nitrofurantoin (S), oxacillin/penicillin (R), tetracycline (S), bactrim (R), vancomycin (S). The patient was started on vancomycin on hospital day two and dexamethasone 0.1 mg/kg q6 × 4 doses.

Additional laboratory examinations showed an elevated C-reactive protein (CRP) 29.8 mg/L (0.0–5.0 mg/L), erythrocyte sedimentation rate (ESR) 5 mm/h (0–10 mm/h), procalcitonin 0.7 ng/mL; DIC panel: protime 12.2 sec (9.4–12.6 sec), international normalized ratio 1.1, fibrinogen 324 mg/dL (140–420 mg/dL), partial thromboplastin time 35.7 sec (21.3–31.3 sec), Bordetella pertussis PCR was negative, Enterovirus PCR was negative, viral cultures were negative. Clindamycin 45 mg/kg/day divided q6h was added on hospital day four for better tissue penetration after excluding endocarditis and meningitis.

The patient gradually recovered, regained his voice, and the mouth, groin, and glans lesions were healed. Treatment consisted of a 14-day course of vancomycin and 9 days of clindamycin. The patient continues to do well with no MRSA recurrence.

## Discussion

Unlike older children who present with stridor and respiratory distress with epiglottitis, the presentation may be different in neonates [6]. Epiglottitis is an inflammation of the supraglottic structures including the vocal cords, which causes these structures to become stiff with decreased mobility often causing aphonia. Initial antibiotic therapy for epiglottitis typically is directed toward the most commonly recognized pathogen, nontypeable *Haemophilus influenzae*, and consists of a third-generation cephalosporin such as ceftriaxone or cefotaxime, a second-generation cephalosporin such as cefuroxime, or the combination of high-dose ampicillin and a β-lactamase inhibitor. The patient was empirically treated with ampicillin, cefotaxime, and vancomycin. Although no direct epiglottis cultures were obtained, we make a case for MRSA as the etiology because of the concomitant MRSA-positive groin and mouth lesions and urine culture. Any of these infectious sources may have caused a transient bacteremia seeding the airway, leading to epiglottitis.

There are case reports of community-associated MRSA (CA-MRSA) strains in well-baby nurseries causing pustular-vesicular lesions in the groin [7], [8]. Superficial phlebitis of greater saphenous vein and lesser saphenous vein have also been associated with CA-MRSA strains, which release toxins causing smooth muscle spasm and aggregation of platelets and enzymes that interact with fibrinogen, resulting in clotting. Some CA-MRSA strains contain the virulence factor Panton–Valentine leukocidin (PVL), which can cause tissue necrosis [9].

PVL is a potent dermonecrotic toxin associated with increased severity of infection and septic emboli and is found in 5% of *Staphylococcus aureus* strains [10], [11]. The MRSA strain found on the patient was not typed to determine if it was community or hospital acquired, and a sample was not sent to confirm that PVL toxin was present; however, the susceptibility pattern was consistent with CA-MRSA. Health-care providers should include epiglottitis in the evaluation of a neonate with aphonia and be aware of the local prevalence of CA-MRSA [12]. Empiric treatment of severe infections with suspected *Staphylococcus aureus* should include both vancomycin and an antistaphylococcal penicillin while awaiting culture and sensitivity results.
